# A Fasciclin-Like Arabinogalactan-Protein (FLA) Mutant of *Arabidopsis thaliana*, *fla1*, Shows Defects in Shoot Regeneration

**DOI:** 10.1371/journal.pone.0025154

**Published:** 2011-09-22

**Authors:** Kim L. Johnson, Natalie A. J. Kibble, Antony Bacic, Carolyn J. Schultz

**Affiliations:** 1 ARC Centre of Excellence in Plant Cell Walls and Plant Cell Biology Research Centre, School of Botany, University of Melbourne, Melbourne, Australia; 2 ARC Centre of Excellence in Plant Cell Walls, Waite Research Institute, The University of Adelaide, Waite Campus, Glen Osmond, Australia; 3 School of Agriculture and Wine, Waite Research Institute, The University of Adelaide, Waite Campus, Glen Osmond, Australia; Purdue University, United States of America

## Abstract

**Background:**

The fasciclin-like arabinogalactan-proteins (FLAs) are an enigmatic class of 21 members within the larger family of arabinogalactan-proteins (AGPs) in *Arabidopsis thaliana*. Located at the cell surface, in the cell wall/plasma membrane, they are implicated in many developmental roles yet their function remains largely undefined. Fasciclin (FAS) domains are putative cell-adhesion domains found in extracellular matrix proteins of organisms from all kingdoms, but the juxtaposition of FAS domains with highly glycosylated AGP domains is unique to plants. Recent studies have started to elucidate the role of FLAs in *Arabidopsis* development. FLAs containing a single FAS domain are important for the integrity and elasticity of the plant cell wall matrix (FLA11 and FLA12) and FLA3 is involved in microspore development. FLA4/SOS5 with two FAS domains and two AGP domains has a role in maintaining proper cell expansion under salt stressed conditions. The role of other FLAs remains to be uncovered.

**Method/Principal Findings:**

Here we describe the characterisation of a T-DNA insertion mutant in the *FLA1* gene (At5g55730). Under standard growth conditions *fla1-1* mutants have no obvious phenotype. Based on gene expression studies, a putative role for FLA1 in callus induction was investigated and revealed that *fla1-1* has a reduced ability to regenerate shoots in an *in vitro* shoot-induction assay. Analysis of FLA1p:GUS reporter lines show that *FLA1* is expressed in several tissues including stomata, trichomes, the vasculature of leaves, the primary root tip and in lateral roots near the junction of the primary root.

**Conclusion:**

The results of the developmental expression of *FLA1* and characterisation of the *fla1* mutant support a role for FLA1 in the early events of lateral root development and shoot development in tissue culture, prior to cell-type specification.

## Introduction

Arabinogalactan-proteins (AGPs) are implicated in several roles in plant growth and development. Of major interest is their putative involvement in cell fate, somatic embryogenesis and cell proliferation, reviewed in [1], [2], [3]. AGPs are highly glycosylated proteoglycans located in the plant cell wall, plasma membrane and many extracellular secretions. Classical AGPs and AG (arabinogalactan)-peptides can be considered the basal form of AGPs in that they have no other domains that might confer functions; as such, the entire protein backbone is proposed to act as a glycosylation scaffold.

**Figure 1:**
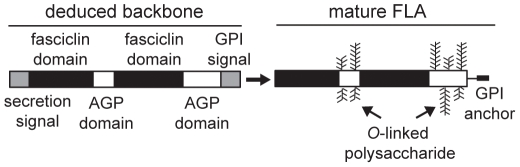
Schematic of a representative FLA containing two AGP domains and two fasciclin domains before and after post-translational modifications. Deduced proteins include an N-terminal secretion signal, two fasciclin domains, two AGP domains and a C-terminal signal sequence for addition of a GPI-anchor. Mature FLAs are predicted to be extensively modified post-translationally with peptidyl proline (Pro) modified to hydroxyproline (Hyp), the addition of *O-*linked oligo/poly-saccharide chains to Hyp residues, and the addition of a C-terminal GPI-anchor.

Many of the AGPs involved in development are chimeric in that their protein backbones have an AGP domain and another domain, such as either a lipid binding [4] or fasciclin (FAS) domain [5], [6]. FLAs are a distinct subclass of AGPs that, in addition to AGP motifs, have fasciclin-like domains [5], [7], [8]. Within the twenty-one genes encoding FLA protein backbones identified in *Arabidopsis* (hereafter referred to as *FLA* genes), a number of subclasses (A, B, C and D) were defined [5]. FLAs can consist of one or two AGP domains and one or two fasciclin-like domains. FLA1 [5], [7] and FLA4 [9] are examples of FLAs with two AGP domains and two fasciclin domains, and are predicted to be extensively modified post-translationally (Fig. 1). In other eukaryotic systems protein-protein interactions of fasciclin-like domains facilitate cell adhesion [10], [11], [12], [13]. Cell adhesion can be broadly defined to include cell-cell and cell matrix interactions [14]. Therefore FLAs are candidate molecules for cell-matrix adhesion because they contain domains with the potential for protein-protein interactions (fasciclin) and domains for protein-carbohydrate interactions (AG) and are located at the cell surface. Fourteen of the *Arabidopsis* FLAs are predicted to be glycosylphosphatidylinositol (GPI)-anchored, due to the presence of a C-terminal hydrophobic signal sequence [5], [15]. Experimental evidence, phosphatidylinositol-specific phospholipase C susceptibility, was provided for the GPI-anchoring of FLA1, FLA7, FLA8 and FLA10 in callus cells [16] and FLA3 and FLA14 in pollen [17]. With so many and varied members in this gene family it is difficult to accurately pinpoint the role of these proteins.

The publicly available *Arabidopsis* DNA insertion lines has made it possible to identify tags in or near many AGP and FLA genes [7], [18]. As is the case for many other multigene families [19], [20], [21], it has been difficult to identify phenotypes for *agp* and *fla* mutants. Consistent with this, the first AGP mutants isolated, *agp17* [22], [23], *agp30* [24] and *fla4* [9] were conditional mutants. For example, *fla4* is salt overly sensitive (*sos5*) [9]. The small number of AGP mutants with phenotypes and the restricted nature of each phenotype highlights the challenge of determining gene function in large gene families, and was recently reviewed [2].

The developmental roles of FLAs in class A, with a single AGP domain and a single FAS domain, has begun to be elucidated due to their specific expression patterns. FLAs 11 and 12 are important for the integrity and elasticity of the plant cell wall matrix as *fla11/fla12* double mutants plant have altered stem biomechanics, and there are changes in the molecular composition and architecture of the stem cell walls [6]. Additionally, the pollen specific *FLA3* gene is shown to have a role in microspore development in *Arabidopsis* with *FLA3*-RNA interference plants having abnormal pollen grains and ectopic expression resulting in fertility defects [25].

We have previously investigated the significant increase in gene expression of several FLAs (confirmed by RNA gel blot analysis) during the *in vitro* production of new shoot and root meristems [5]. In *Arabidopsis* a two-step (indirect procedure) is used in which plant explants are induced to form callus on high auxin:low cytokinin, callus inducing medium (CIM) and then transferred to shoot inducing medium (SIM), containing low auxin:high cytokinin, to induce shoots [26]. *FLA1* is one of many genes whose expression changes when cells proliferate to produce callus [5], [27].

Callus was long considered to be an undifferentiated tissue due to its high regeneration ability and seeming unorganized structure. Recent studies have shown that callus formation is not a process of reprogramming to an undifferentiated state as previously believed, but rather the differentiation of pericycle-like cells toward root meristem-like tissue [28], [29], [30]. This was shown with elegant expression studies of genes involved in lateral root development and pericycle identity, as well as evidence that mutant plants incapable of lateral root initiation are unable to form callus from a number of tissues. These studies have provided a new understanding of the identity of callus and invite further studies into how these root-like cells initiate development of new shoots.

In this paper we investigate whether *FLA1* plays a role in callus and shoot developmental processes using a *fla1* mutant. We show that *fla1-1* has no obvious phenotype under standard growth conditions yet has a reduced ability to regenerate shoots from root explants after CIM and SIM treatment. A *FLA1p*:GUS reporter shows expression in the root tip and lateral roots similar to auxin reporters involved in lateral root initiation. Based on the developmental expression of *FLA1* and the phenotype of the *fla1-1* mutant, we propose a role for FLA1 in shoot development and formation of lateral roots.

## Results

### Identification and characterisation of plants with an insert in *FLA1*

**Figure 2:**
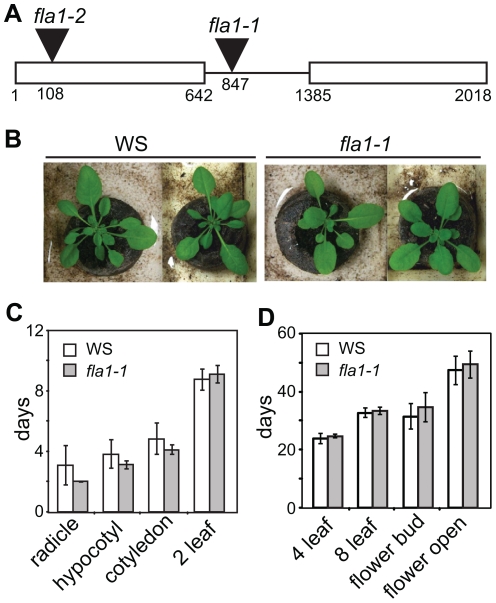
Characterisation of DNA insertion mutants for *FLA1*. A Schematic representation of the T-DNA insertion locus (black triangle) of *fla1-1,* located in the intron of *FLA1* identified by screening the Feldmann mutant lines (WS ecotype), and *fla1-2,* located in exon 1 (SALK insertion mutant, COL ecotype). B Phenotypic comparison of six-week-old WS and *fla1-1* mutant *Arabidopsis* plants grown in soil. Appearance of key developmental stages (days) of seedlings on plates (C) and pots (D) according to Boyes et al. [32].

A T-DNA insert was identified in the intron of *FLA1* (Fig. 2A) by screening the Feldmann T-DNA lines available from the ABRC using a PCR method [31]. Homozygous mutants (*fla1-1)* were identified by PCR and confirmed as a knockout mutant using RNA gel blot analysis (Fig S1). Segregation analysis on kanamycin selective media and DNA gel blots indicated this line has only one T-DNA insert (data not shown).

Homozygous mutant *fla1-1* plants were grown under standard conditions to determine if they differed from wild-type (WS ecotype) plants. No obvious growth phenotypes were observed in *fla1-1* mutants compared to wild-type (Fig. 2B). A more detailed analysis was performed based on the stages and growth descriptions outlined in Boyes et al. [32]. As many of the later stages of growth, such as leaf size and plant height, are highly variable, the most reliable growth stages were used in this analysis [32]. Comparison of *fla1-1* and wild-type seedlings during 14 d growth on plates showed no significant difference in rate of shoot growth (Fig. 2C). For the soil-based assay rosette growth and two key stages of flower development (appearance of the first inflorescence meristem and opening of the first flower (stage 13 [33]) were compared (Fig 2D). No significant differences were observed between wild-type and *fla1-1* mutants (Fig. 2C, D).

### Response of the *fla1-1* mutant to shoot development

**Figure 3:**
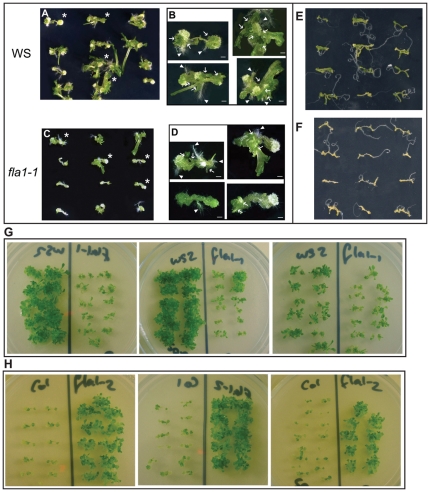
Shoot induction phenotypes of *fla1* mutants. A-D Wild-type and *fla1-1* mutant root explants after 4 d treatment on callus induction medium (CIM) then 14 d incubation on shoot induction medium (SIM). (A and B) Wild-type (WS) and (C and D) *fla1-1* mutant root explants. (A and C). Root explants from the zone of maturation of the primary root where lateral roots were forming were incubated on CIM then transferred to SIM. (B and D) Four representative root explants (indicated on (A) and (C) by an asterisk) from the zone of maturation of wild-type (B) and *fla1-1* mutant (D) roots after CIM and SIM treatment. The long arrows indicate the base of a new shoot and the arrowheads indicate the tip of a new root. 13 independent experiments comparing WS and *fla1-1* were performed. (E) Wild type and (F) mutant root segments after only 1 d on CIM, followed by 14 d SIM. (G) Wild-type (WS) and *fla1-1* mutant root explants after 4 d on CIM then 14 d SIM. (H) Wild-type (COL) and *fla1-2* mutant root explants after 4 d on CIM then 14 d SIM. Experiments comparing COL and *fla1-2* were repeated three times, and WS2 and *fla1-1* were also compared in each of these three experiments. Scale bar is 1 mm.

In an attempt to uncover a phenotype for *fla1-1*, a directed approach was used based on the significant increase in *FLA1* mRNA based on RNA gel blot analysis of tissue during callus and shoot induction experiments [5]. To determine if FLA1 was important for shoot development, *fla1-1* mutants and wild-type plants were tested for their ability to produce new shoots and roots from callus. *Arabidopsis* roots, from the zone of maturation (region containing root hairs), from 14 d mutant and wild-type plants were cut into explants, transferred first to CIM and then to SIM [34]. After 4 d CIM and 14 d SIM treatment the *fla1-1* mutant tissue had a reduced number of green foci and shoots compared to wild-type (Fig. 3A, C and Table 1). New shoots were scored based on i) leaf or root like morphology that was > 1 mm in length and ii) a distinct emergence point from the root, such that if multiple leaf-like projections were growing from a major callus, this was only counted as one shoot. Some root explants of *fla1-1* could still form shoots (Fig. 3C, D), however, a significant reduction in the formation of both shoots (≈ 49%) and roots (≈ 15%) was consistently observed (Table 1). Some variation is seen in the number and size of shoots between experiments, but the difference between wild-type and mutant is always apparent from the colour of the tissue (yellow vs green) without the need to count new shoots. The yellow vs green difference is clearly shown in Fig 3E, F, but is not always apparent in photographs (Fig 3 A, C).

**Table 1:** Shoot and root regeneration of wild-type and *fla1-1* mutants.

	Shoots	Roots
wild-type	2.3±0.4	4.3±0.3
*fla1-1*	1.1±0.2 *	3.7±0.3 *

The average number of shoot (or root)-like growths per root segment (± standard error), that have a distinct point of emergence from the callus and are greater than 1 mm in length. The data were collected from several different plates (after CIM and SIM treatment), and a total of 109 shoot-like projections were counted for each of wild-type and mutant tissue. The asterisk indicates a statistically significant difference between wild-type and *fla1-1* (t-test; P<0.05).

To determine how early in the shoot induction process FLA1 is required we reduced the time of CIM treatment. Wild-type and *fla1-1* mutant root explants were incubated on CIM for 1, 2, 3 or 4 d then transferred to SIM for 14 d. One day on CIM, followed by 14 days on SIM, is all that is required to observe a difference between *fla1-1* mutant and wild type tissue (Fig. 3E, F).

An additional *fla1* allele was obtained to determine if a defect in callus initiation was also observed. The *fla1-2* allele was identified in the SALK lines (SALK_058964) [35] and contains a T-DNA insert in the first exon, 108 bp downstream of the ATG (Fig. 2A). The *fla1-2* allele is a knockout mutant as shown by RNA gel blot analysis (Fig. S1). This allele exists in the Columbia (COL) ecotype and the ability to form callus was compared. The COL ecotype showed a decreased ability to regenerate shoots compared to the WS ecotype and the *fla1-2* allele showed a similar number of shoots to the WS ecotype (Fig. 3G, H). Differences in the ability of Arabidopsis ecotypes to form callus has been reported and the regeneration capacity for both WS and COL varies depending on the experimental conditions [29], [36]. It is possible that ecotype specific regulatory factors, such as different promoter elements and/or differential transcription factors, are involved in shoot regeneration and the loss of *FLA1* gene produces different phenotypes in different genetic backgrounds dependent on these factors (see Discussion).

### Light microscopy analysis of callus and shoot induced tissue

**Figure 4:**
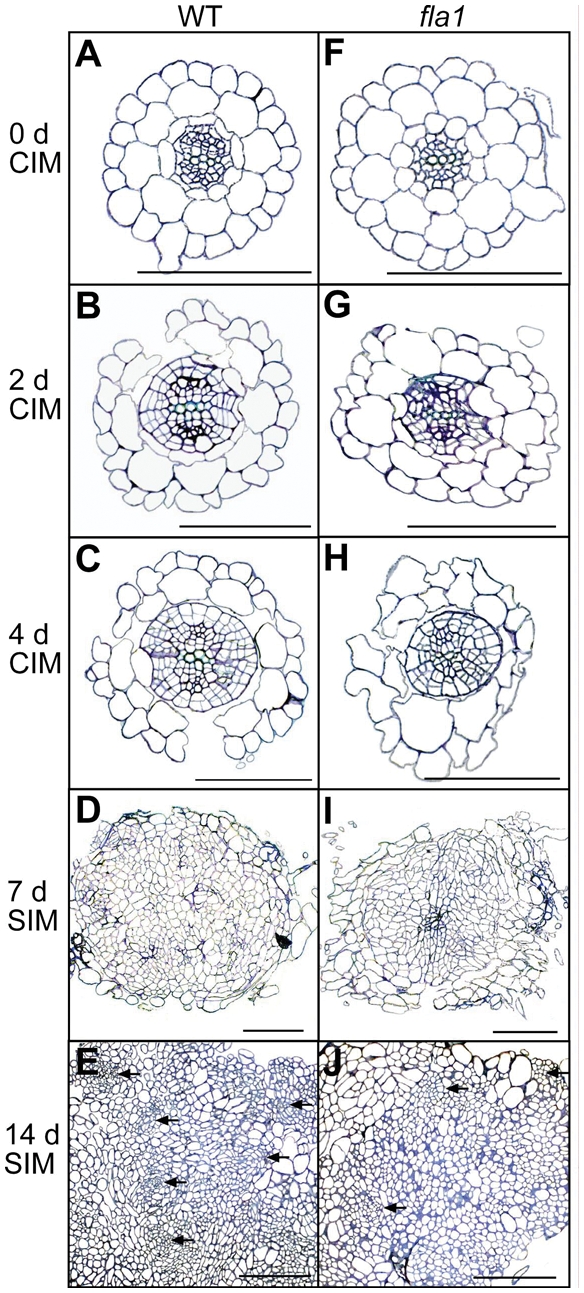
Light microscopy of wild-type and *fla1-1* root explants after CIM and SIM treatment. Sections through a region of WS (A to E) and *fla1-1* (F to J) root explants. Transverse sections through the differentiated zone of a 14 d wild-type (A) and *fla1-1* mutant (F) root. Transverse sections through root explants incubated on CIM for 2 d (B and G) and 4 d (C and H). Wild-type (D and E) and *fla1-1* mutant (I and J) root explants after 4 d CIM and 7 d (D and I) or 14 d (E and J) SIM treatment. Centres of radial organisation which are presumptive sites of meristem formation are indicated by arrows. Sections were stained with toluidine blue. Scale is 0.1 mm; P, pericycle.

Toluidine blue stained tissue sections of wild-type WS and *fla1-1* root explants after 0, 2, 4 d CIM and 7 and 14 d SIM treatment were analysed by light microscopy to examine cell structure and organisation (Fig. 4). During CIM treatment the pericycle cells divide, expanding the diameter of the vascular bundle [27]. The pericycle cells in both the wild-type and *fla1-1* mutant roots have undergone division after 2 d, however after 4 d CIM treatment the *fla1-1* mutant has fewer divisions (compare Fig. 4B and C with Fig. 4G and H). After CIM and SIM treatment the root segments from *fla1-1* mutants appeared as a disorganised mass of cells, as did the wild-type root segments, however, the mutants showed fewer dense centres of radial organisation (see arrows, Fig. 4D, E and J) which are thought to be sites of presumptive meristem formation [27]. These results indicate *FLA1* may be acting in the early stages of re-differentiation.

### *fla1* mutants show increased numbers of lateral roots

**Figure 5:**
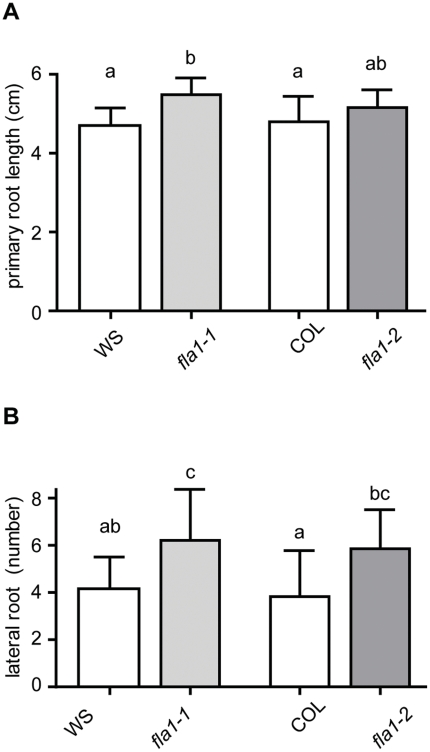
Comparison of root phenotypes of *fla1* mutant alleles in different genetic backgrounds. A Length of primary root (cm) of wild-type (WS), *fla1-1,* wild-type (COL) and *fla1-2*. Significance levels a and b (P<0.01) are based on Tukey's post test (1-way analysis of variance). B Number of lateral roots of wild-type (WS), *fla1-1,* wild-type (COL) and *fla1-2*. Number of seedlings analysed was n = 12, 14, 13, 14 for WS, *fla1-1,* COL, *fla1-2* respectively. Significance levels a and c (P<0.01), others (P<0.05), are based on Tukey's post test (1-way analysis of variance). Error bars represent standard deviation of the mean.

Shoot regeneration has many features in common with lateral root primordia formation [28], [29], [30]. To determine if FLA1 also plays a role in lateral root formation we analysed the number of lateral roots in *fla1* mutants and wild-type. Both *fla1-1* and *fla1-2* alleles showed a small but significant increase in the number of lateral roots compared to the respective wild-types (Fig. 5B). In addition, the length of the primary root was also slightly increased in both *fla1* mutants (Fig 5A). To gain further understanding of the role of FLA1, both in root and shoot development, we fused the *FLA1* promoter from the WS ecotype to the GUS reporter gene (*FLA1*p:GUS).

### *FLA1* expression is developmentally regulated

**Figure 6:**
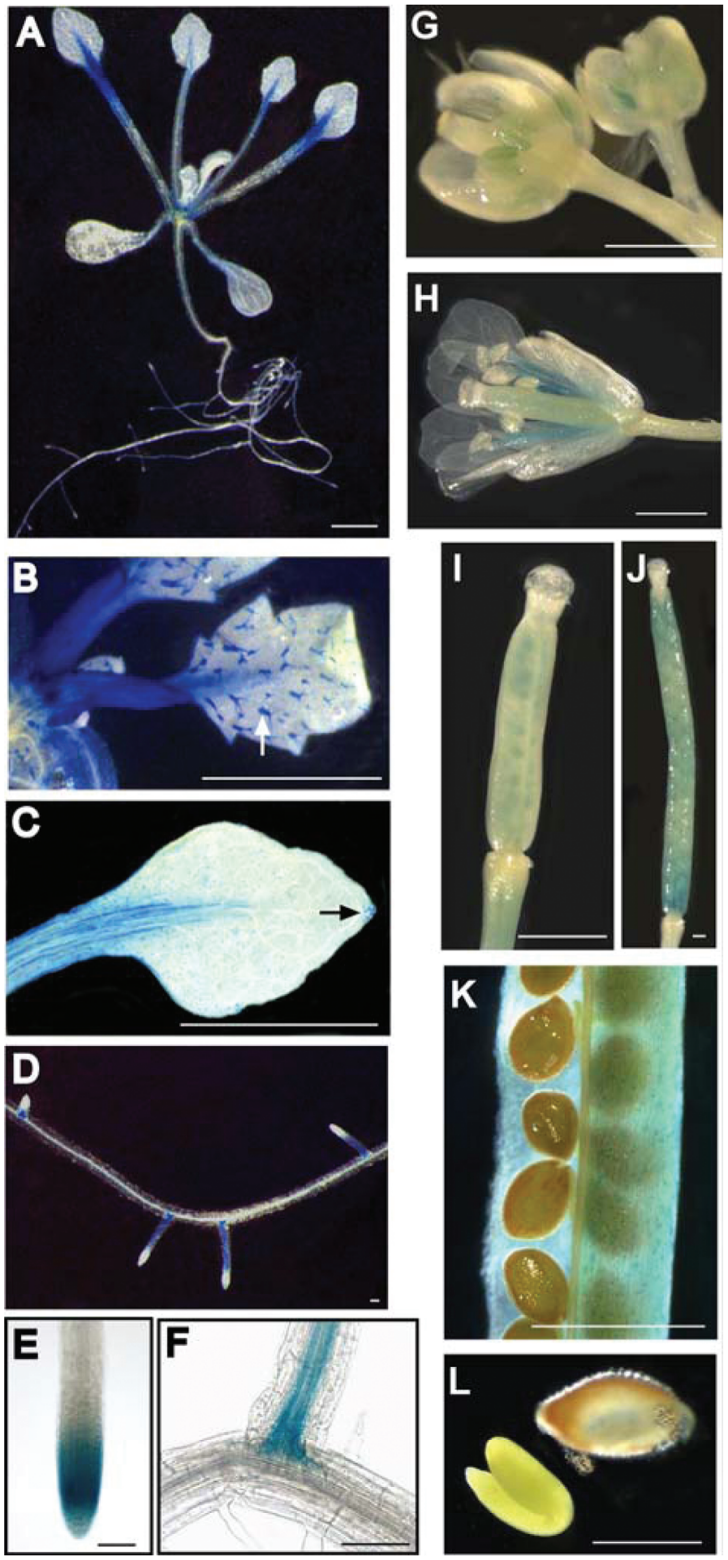
*FLA1* promoter:GUS analysis of 14 d plate grown seedlings and flowering, soil grown *Arabidopsis* plants. GUS activity was detected in the A petioles and roots. B petioles and base, stomata and leaf hairs (trichomes, indicated by a white arrow) of young leaves. C stomata throughout the leaf and at the hydathode at the leaf tip (indicated by a black arrow). D lateral roots. E primary root, but only in the early elongation zone of the root tip. F developing lateral roots in the vascular and pericycle cells. G developing anthers of closed flowers. H stamen filaments (but not anthers) of open flowers. I early embryos of fertilised stigma and style, dissected from an open flower. GUS expression was observed in vegetative portion of developing siliques (J) including the stomata (K) and not in embryos (L). Scale bar is 0.1 mm, except in (C) where it is 0.03 mm.

The expression pattern of *FLA1* was investigated using *FLA1*p:GUS lines. Six independent lines were analysed in the T_2_ generation to check for consistency of GUS staining in the major tissues of seedlings (14 d) and mature plants (6-week old). One representative line is shown in detail. In the shoots of seedlings, GUS activity was visible in the petiole, in stomata and trichomes (Fig. 6A to C). In flowers *FLA1*p:GUS expression appears to be developmentally regulated (Fig. 6G to L). Expression was detected in the developing anthers of closed flowers and in the stamen filaments, but not in the anthers of open flowers (Fig. 6G and H). After fertilization, GUS activity was seen in the early embryos in the pistil (Fig. 6I). In contrast, in developing siliques, staining was detected in the vegetative portion (seed pod) and not mature embryos (Fig. 6K, L). Weak GUS staining was observed in the inflorescence stem (data not shown).

In roots, expression was detected in the mature vasculature of lateral roots and the elongation zone of the primary root, but absent from most of the primary root (Fig. 6D to F). In summary, *FLA1*p:GUS shows a developmentally regulated expression pattern in discrete tissues of the leaf petiole, stomata and trichomes, anthers and early embryos in flowers and primary root tip and lateral root primordia.

As callus initiates from a similar pathway to lateral root initiation the expression of *FLA1*p:GUS was investigated further. We previously investigated the up-regulation of *FLA1* expression in root tissue after callus and shoot induction assays using RNA gel blots [5]. To provide more detailed analysis of the cell type-specific expression of FLA1 during this process, the *FLA1*p:GUS reporter was used.

### FLA1p:GUS expression during callus initiation and shoot development

**Figure 7:**
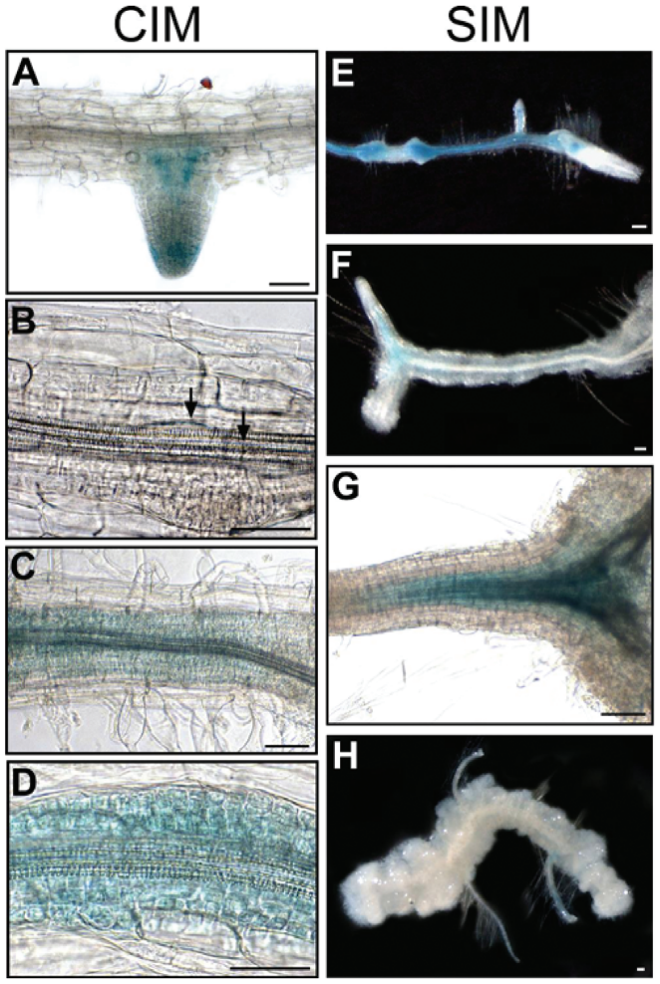
*FLA1* promoter:GUS analysis of *Arabidopsis* root explants after CIM and SIM treatment. GUS activity was detected in the A lateral roots of 14 d *Arabidopsis* seedlings before incubation on CIM and SIM (see also Fig. 6). B some vascular and pericycle cells in root explant after 2 d CIM. C along the length of the root explant in the vasculature and de-differentiating pericycle cells after 3 d and 4 d (D) CIM treatment. E in callus and vascular tissue of root explant after 4 d CIM and 3 d SIM. (F and G) in the vascular tissue and pericycle cells of newly forming roots after 4 d CIM and 7 d SIM. H in the vascular tissue of newly formed roots of root explants after 4 d CIM and 14 d SIM. Scale bar is 0.1 mm.

To characterise the regulation of *FLA1* during root re-differentiation and shoot development in tissue culture, GUS activity was analysed in root explants after 0, 2, 3 or 4 d CIM and 3, 7, and 14 d SIM treatment (Fig. 7). Before CIM treatment (0 d) GUS expression is seen only in the lateral roots of 14 d seedlings (Fig. 7A) whereas after 2 d CIM treatment it was visible in some vascular and pericycle cells of the root explant (Fig. 7B). After 3 and 4 d CIM treatment GUS expression was seen in large sections of the root explant and lateral roots, throughout the vascular tissue and in expanding pericycle cells (Fig. 7C, D). After 3 d SIM treatment GUS expression was present in callus and vasculature of the root explant (Fig. 7E) and after 7 d on SIM GUS staining was no longer seen along the length of the root explant and appears in the pericycle and vascular bundle of newly forming roots (Fig. 7F, G). GUS expression was evident in the vascular tissue of newly formed roots after 14 d SIM (Fig. 7H). The up-regulation of *FLA1* transcripts [5] and *FLA1*p:GUS expression during CIM treatment suggests FLA1 may be involved in initial stages of callus formation in the lateral root initiation pathway.

## Discussion

### *fla1-1* mutants have a role in shoot regeneration

We have investigated the role of a Fasciclin-Like Arabinogalactan-protein (FLA1) during development. The expression of *FLA1* in lateral roots, callus (CIM) and shoot induction (SIM) experiments [5], and the *fla1-1* mutant phenotype is consistent with FLA1 having a role in lateral root development and shoot regeneration from root tissue.

After treatment on CIM a large number of pericycle cells re-differentiate and show expression of lateral root initiation and root tip meristem factors [29], [30]. The increase in FLA1p:GUS activity on CIM media (Fig. 7), is consistent with the dramatic increase in RNA expression of untreated roots compared to root segments treated with CIM as previously shown by RNA gel blot analysis [5]. *FLA1* is also one of many genes whose expression changes when cells proliferate to produce callus [34]. During preincubation of CIM, root segments are thought to progressively acquire competence to respond to shoot induction signals. The *fla1-1* mutants are still capable of forming roots, green foci and shoots after CIM and SIM treatment yet show a reduced number compared to WS wild-type ecotype (Fig. 3D, G, Table 1). The phenotypic difference in *fla1-1* mutants compared to wild-type after only a relatively short incubation on CIM (Fig. 3E, F) suggests that FLA1 may be important early in this process. It cannot be excluded that FLA1 is required for shoot development rather than initial stages of re-differentiation, however, we propose that FLA1 is more likely involved in the first stage of competence acquisition based on 1) its expression is up-regulated most significantly at this stage, 2) changes in the length of CIM treatment results in changes in amount of shoot regeneration and 3) differences in the number of lateral roots.

It has been proposed that a ‘repressed state’ is overcome by CIM treatment [37]. This state likely represents the majority of the pericycle cells remaining in the G1 phase of cell division, whereas those that originate opposite the xylem poles and initiate lateral root primordia, (and are capable of shoot development in the absence of CIM), advance to the G2 stage [38]. The increased expression of *FLA1p*:GUS in pericycle cells after CIM treatment suggests it may be one of the components de-repressed during this process to enable shoot development pathways to be initiated. It is likely that *FLA1* is regulated in an auxin-dependent pathway as primary hormone response genes, such as Aux/IAA genes, are up-regulated during incubation on auxin-rich CIM. Regulation of *FLA1* by cytokinin cannot be ruled out and may have an inhibitory effect on *FLA1* expression as indicated by the reduced *FLA1*p:GUS expression patterns in SIM treated explants. Further experiments to validate the potential regulation of *FLA1* in auxin- and cytokinin-dependant pathways remain to be investigated and will be important for understanding the role of FLA1.

Differential regulation of *FLA1* in different ecotypes (http://www.ebi.ac.uk/arrayexpress/) may explain the varied response of *fla1* alleles to callus induction assays. Opposite effects on shoot regeneration capacity has also been reported for different mutant alleles of A-type *ARABIDOPSIS RESPONSE REGULATOR, arr15*, suggesting the type of mutation and the experimental conditions may play a role [39], [40]. Depending on the conditions used both COL and WS ecotypes have been reported to have poor regenerative capacity [29], [36]. It will be interesting to determine if *FLA1* expression levels and developmental regulation differs between the two ecotypes. Alternatively, the regulation of other *FLAs* or unrelated genes in the same developmental pathway could be regulated differently in the different ecotypes. Complementation experiments of *fla1-1 (WS)* and *fla1-2 (COL)* mutants with native gene constructs (eg *FLA1p(WS):FLA1(WS)*) compared to promoter swap experiments (*FLA1p(Col):FLA1(WS)* and *FLA1p(WS):FLA1(COL))* are needed to confirm the involvement of *FLA1* in the different ecotypes. Other areas for further research include a search for differences between the WS and COL ecotypes in the regulatory elements in *FLA1* promoters as well as the differential expression of transcriptions factors, such as *ESR1 and 2* [41], and *WIND1* [42] that have a demonstrated role in shoot regeneration. Investigation of the role of FLA1 in shoot regeneration in a number of different ecotypes is an intriguing area for further research to dissect the natural variation in FLA1 function.

### Determining the role of FLA1 during development

Large arrays of genes are differentially regulated during both CIM and SIM treatments. A number of global gene expression studies have been undertaken to identify genes involved in shoot development from callus tissue [28], [30], [37]. The general patterns of gene expression were: (1) up-regulation of a number of hormone response genes, largely Aux/IAA genes, during preincubation on CIM, (2) induction of many genes that encode signalling and/or transcription components before shoot emergence at the approximate time of shoot commitment and (3) as shoots emerged, genes that encode products of differentiated cells, mostly genes that encode components of the photosynthetic apparatus, were highly induced [28]. Detailed investigation by Sugimoto et al. [30] of the initial callus induction stage revealed that callus resembles a root-like tissue in transcriptional profiles and studies of reporter genes involved in lateral root primordia development have been shown to be expressed in the early stages of callus formation [29], [37]. Interestingly *FLA1* is expressed in both the root meristem and lateral root tissues (Figs 6D-F & 7) and is reminiscent of the cellular distribution of auxin [43], [44]. The decreased ability of *fla1-1* to form callus and shoots in culture may therefore indicate FLA1 normally functions in roots. Additionally both *fla1* mutant alleles show a small increase in the number of lateral roots and root length compared to wild-type. It is unclear how loss of FLA1 may result in more lateral roots and will require further studies to address if this is related to changes in lateral root initiation or emergence.

### What is the mechanistic role of FLAs in the cell wall?

The proposed function of FLA1 in acquisition of competence and the occurrence of *FLA1*p:GUS activity in the elongation zone of the primary root and lateral roots may be related to a role in cell identity. Antibodies that recognise carbohydrate epitopes on AGPs show tissue-specific and spatio-temporal appearance of AGPs that correlate with certain aspects of plant development [45], [46], [47], [48]. This has led to proposed functions for AGPs in defining cell identity or cell fate, reviewed in [1], [49], [50]. In carrot suspension cultured cells, AGPs recognised by the JIM8 antibodies are polarised in cells about to divide resulting in different fates of the daughter cells [51]. Anti-FLA1 antibodies will be necessary to confirm the plasma membrane location of FLA1 and to determine if FLA exhibit polarity in cells. Attempts to generate polyclonal antibodies specific for FLA1 are currently being undertaken.

Development of a new organ (such as a shoot or lateral root) requires the early establishment of an auxin gradient and this is achieved through polarised targeting of important proteins such as PIN and AUX1, and controlled cell expansion with proteins such as COBRA in roots, reviewed in Fischer et al. [52]. An intriguing new finding is the regulation of PIN1 localization by cellulose [53]. Recent studies indicate FLA11 and FLA12 may affect cellulose deposition [6] and investigation of GPI-anchored AGPs suggest they are secreted to the cell surface with cellulose synthase [3]. Investigation of the polarity of PIN1 in *fla1* mutants will be an interesting avenue for further study as defects in cell expansion have been observed for the *fla4* mutant that is salt overly sensitive (*sos5*/*fla4*).

Loss of active FLA4 in the elongation zone of the root permits radial expansion which is normally inhibited to allow longitudinal expansion [9]. *FLA4/SOS5*, is highly expressed throughout the vasculature and cortex of all roots and relatively weakly in epidermal cells and root hairs [9]. The absence of *FLA1*p:GUS activity in the cortex and epidermal cells could explain why *fla1-1* mutants do not have the root swelling phenotype of *fla4/sos5* (data not shown). Expression of other FLAs in tissues where *FLA1*p:GUS activity was detected, such as guard cells, trichomes and petioles, could explain why dramatic phenotypes were not observed in these tissues. Multiple double mutant combinations and targeted physiological experiments relevant to each tissue type are needed to fully understand FLA function.

A proposed model for FLA function in the extracellular matrix suggests FLAs interact through their fasciclin domains, most likely by non-covalent interactions [54], to either control or limit cell expansion prior to cross-linking of cell wall polysaccharides. It is possible the FLAs are the AGPs that co-localize with wall-associated kinases (WAKs) at vertices (foci) of the polyhedral array near the plant cell surface known as the plasmalemmal reticulum, that links the cytoskeleton-plasma membrane and cell wall [55], [56]. Loss of FLAs would lead to increased cell expansion in an inappropriate direction.

One possibility is that FLA1 is involved in regulating cell expansion in the newly formed lateral root. Degradation of the cell walls in the cells adjacent to the lateral root is required to allow emergence and is known to be regulated by auxin [38]. Little is known however about the regulation of cell walls in the lateral roots themselves, the expansion of which must be tightly coordinated. It will be essential to learn more about the complex interactions of cell wall components in order to define the role of FLAs in development.

## Materials and Methods

### Identification of *fla1* mutants

Pools of DNA from the Feldmann T-DNA lines [31] were ordered from the Arabidopsis Biological Resource Centre (ABRC, stock # CD5-7). Each pool of DNA was screened with the forward and reverse gene-specific primers and left border insert specific primers (FLA1-F1; 5′-AACCAAACTCTTCACTCTCTCCAACAATG-3′, FLA1-R1; 5′-AGTCGCATATATAGCTAAAGGCTGCTCAT-3′, LB; 5′-ATGTGTAAATATTGCGCGGAGTCATTACA-3′). The seed stock corresponding to the *fla1* insertion line (stock # CS01810) and the background ecotype for generation of the Feldmann T-DNA lines, Wassilewskija-2 (WS, stock # CS2360), were obtained from the ABRC (Columbus, OH). *fla1-2* is a SALK line [35], SALK_058964 (Columbia (COL) background) with a T-DNA insert located in the first exon of FLA1.

### Construction of *FLA1* promoter:GUS plasmids

Constructs for *FLA1* promoter:GUS fusions were created by subcloning the EcoR1/Pst1 fragment containing the GUS gene from pBI101.3 (Clonetec) into the EcoR1/Pst1 site of pGreen 0000 vector (http://www.pgreen.ac.uk/). The promoter region was determined as being from after the polyA addition site of the upstream gene (At5g55740), up to and including the start codon of the *FLA1* gene. The *FLA1* promoter:GUS construct was created by subcloning a 1.5 kb region of the *FLA1* promoter generated by PCR (primers; FLA1, forward: 5′-CAAGAATTGAGAAGCTTTGTGA-3′, reverse: 5′-CATTGTTGGAGAGAGTGAAGAG-3′) using the Herculase enzyme (Stratagene, CA, USA) in a standard PCR protocol. The PCR fragment was cloned into the Sma1 site of the pGreen 0000 GUS plasmid, and sequenced. Constructs were introduced into *Agrobacterium tumefaciens* strain GV3101 containing the binary vector pSoup (http://www.pgreen.ac.uk/).

### Growth conditions of transgenic *Arabidopsis* plants and GUS assays

Wild-type *Arabidopsis* (WS) was transformed using the floral dip method [57]. Primary transformants (6 plants) were screened by PCR using GUS primers (forward; 5′-AGTACTCTGCTGTCGGCTTTAACCTC-3′, reverse; 5′-AATAATCCAGCCATGCACACTGATAC-3′). Selfed seeds from primary transformants selected on Kan were grown for 14 d or 6 weeks and used in GUS activity assays (5 mM ferricyanide, 5 mM ferrocyanide, 42.3 mM NaH_2_PO_4_, 57.7 mM Na_2_HPO_4_, 50 mM EDTA, 0.1% TritonX-100 and 0.25 mg.mL^−1^ 5-bromo-4-chloro-3-indoyl-β-d-glucuronide (X-Gluc)) overnight at 37°C [58].

### Plant material for plate based assays, shoot development and p*FLA1*:GUS analysis

*Arabidopsis* tissue from wild-type (WS) strain CS2360, *fla1-1* mutant and FLA1p:GUS seeds were surface sterilised with 12% (v/v) hypochlorite for 5 min, rinsed with sterile water and transferred in 0.8% SeaPlaque agarose (FMC Bioproducts) to sterile MS plates (1 x MS (GibcoBRL), 3% sucrose, 0.8% agar). Plates were incubated at 4°C for 3 days then placed in a chamber (120 µmol m^−2^.s^−1^) 16 h light, 8 h dark, day temperature 22°C, night temperature 16°C for 10 to 1 d. Seedlings were transferred from plates to peat pellets (Jiffy products international) and grown in growth chambers (21°C, 16 h light: 150 µmol m^−2^.s^−1^) for a further 4 weeks for comparison of *fla1-1* and wild-type plants or 6 weeks for FLA1p:GUS analysis of flowering plants. For lateral root development, sterile seed were placed on media (0.5 x MS + vitamins (PhytoTechnology Laboratories, M519), 1% sucrose, 0.5% MES, pH 5.6, 0.375% phytogel (Sigma)), kept at 4°C for 3 d, then transferred to growth chamber for 7 d (100 µmol m^−2^.s^−1^) continuous light, 22°C.

### Plant material for callus induction and shoot development

Callus induction and shoot development were performed according to the method of Cary et al. [34]. Approximately 10 root sections of 5 to 10 mm were placed onto three replicate plates (1 x Gamborgs basal salt medium (PhytoTechnology Laboratories), 2% sucrose, 0.8% agar, pH 5.2) of callus-induction medium (CIM) containing 2.2 µM 2,4-dichlorophenoxyacetic acid (2,4-D) and 0.2 µM kinetin in a 20°C chamber, 16 h light, 8 h dark. After incubation on CIM for 4 d, root explants were transferred to plates containing shoot induction medium (SIM) containing 0.9 µM 3-indoleacetic acid (IAA) and 5 µM isopentenyladenine (IP) for a further 14 d except where noted otherwise. Photographs of plates were obtained using a Leica DC300F digital camera (Leica) with a macro-switar (1∶1, 1 or 1∶1, 9) lens (Bolex) and individual root segments with a dissecting microscope (Leica) and direct links to IM50 image software (Leica). These images were used in the comparison of *fla1-1* mutant plants to wild-type and for scoring the number of shoots and roots. Shoots and roots were counted based on being greater than 1 mm in length and having a distinct point of emergence from the callus. In some experiments (light microscopy analysis or GUS assays), root segments were collected before transfer to CIM, after 2, 3 and 4 d on CIM and 3, 7 and 14 d on SIM.

### RNA gel blot analysis

RNA gel blot analysis was performed using DIG probes as described previously [8]. Shoot tissue from 8 d seedlings was used, from plants grown and used for CIM/SIM assays. RNA was extracted using TRIZOL reagent (Invitrogen) according to the manufacturer's instructions and 10 µg RNA electrophoresed through a 1% formaldehyde gel. Single-stranded digoxigenin labelled probes were prepared using a two-stage polymerase chain reaction protocol [59], with primers are as follows: FLA1-F, 5′-CTCTCCCTCCACGTCCTTTTAGATTACTT-3′, FLA1-R, 5′- AGTCGCATATATAGCTAAAGGCTGCTCAT-3′, Probe size was 702 nt.

### Light microscopy analysis

WS and *fla1-1* mutant root explants were fixed in 3% paraformaldehyde, 1.25% glutaraldehyde and 0.05 M phosphate buffer. After dehydration the tissue was embedded in LR White hard grade resin (AGAR Scientific Ltd). Tissue sections (2 µm thick) were baked at 65°C onto glass slides (Livingstone) and stained with toluidine blue (0.05% in 0.1 M sodium acetate, pH 4.4) for 2 min. Digital images were obtained using a Leica DC300F digital camera (Leica) with IM50 image software (Leica). The images are representative of sections from 5 independent root segments.

## Supporting Information

Figure S1 **RNA gel blot of shoot tissue from wild-type and** ***fla1*** **mutants.**
(TIF)
