# Supplementary figures and images for: A Fasciclin-Like Arabinogalactan-Protein (FLA) Mutant of Arabidopsis thaliana, fla1, Shows Defects in Shoot Regeneration
Source: PLoS One. 2011 Sep 22;6(9):e25154. doi: 10.1371/journal.pone.0025154 (PMC3178619; doi:10.1371/journal.pone.0025154)

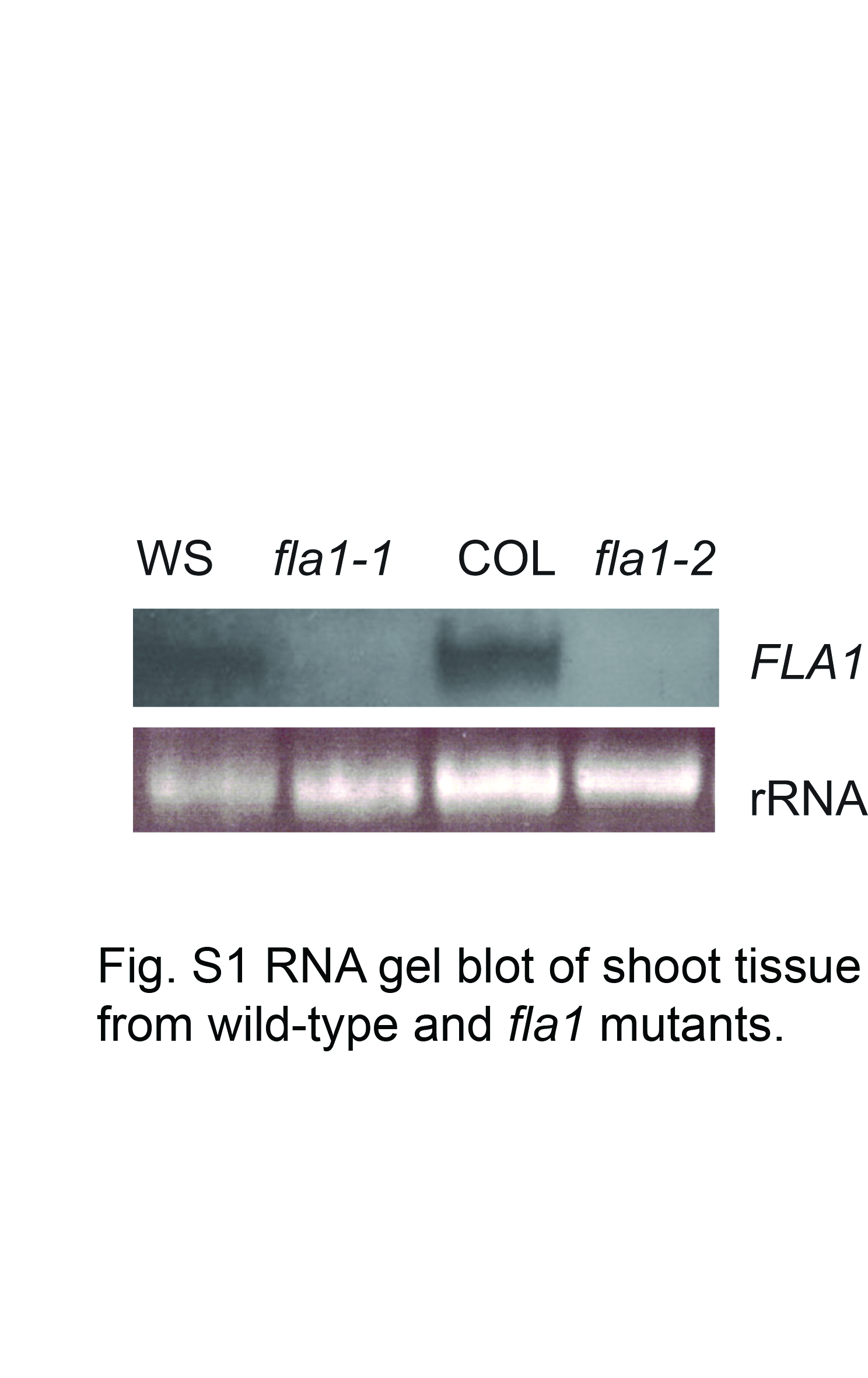

Supplement: Figure S1 — RNA gel blot of shoot tissue from wild-type and fla1 mutants. (TIF) [file pone.0025154.s001.tif]
